# Effects of the Continuous Administration of an *Agaricus blazei* Extract to Rats on Oxidative Parameters of the Brain and Liver during Aging

**DOI:** 10.3390/molecules191118590

**Published:** 2014-11-13

**Authors:** Anacharis B. de Sá-Nakanishi, Andréia A. Soares, Maria R. M. Natali, Jurandir Fernando Comar, Rosane M. Peralta, Adelar Bracht

**Affiliations:** 1 Department of Biochemistry, State University of Maringá, 87020900 Maringá, Brazil; E-Mails: anacharis@bol.com.br (A.B.S.-N.); andasoares7@gmail.com (A.A.S.); jfcomar@uem.br (J.F.C.); rmperalta@uem.br (R.M.P.); 2 Department of Morphological Sciences, State University of Maringá, 87020900 Maringá, Brazil; E-Mail: mrmnatali@uem.br

**Keywords:** aging, oxidative stress, oxidative damage, brain, liver, *A. blazei* treatment

## Abstract

An investigation of the effects of an aqueous extract of *Agaricus blazei*, a medicinal mushroom, on the oxidative state of the brain and liver of rats during aging (7 to 23 months) was conducted. The treatment consisted in the daily intragastric administration of 50 mg/kg of the extract. The *A. blazei* treatment tended to maintain the ROS contents of the brain and liver at lower levels, but a significant difference was found only at the age of 23 months and in the brain. The TBARS levels in the brain were maintained at lower levels by the *A. blazei* treatment during the whole aging process with a specially pronounced difference at the age of 12 months. The total antioxidant capacity in the brain was higher in treated rats only at the age of 12 months. Compared with previous studies in which old rats (21 months) were treated during a short period of 21 days with 200 mg/kg, the effects of the *A. blazei* extract in the present study tended to be less pronounced. The results also indicate that the long and constant treatment presented a tendency of becoming less effective at ages above 12 months.

## 1. Introduction

*Agaricus blazei* is a basidiomycete which has become the subject of great interest due to its nutritional value and pharmacological properties [1,2,3]. In a preceding work we have reported the effects on oxidative and functional parameters of the brain tissue and brain mitochondria of treating old rats during 21 days with an *A. blazei* aqueous extract [4]. In the same series of investigations the effects of the *A. blazei* treatment on the oxidative state of the liver from old rats was equally examined [5]. In general terms, the daily intragastric administration of 200 mg/kg of an aqueous *A. blazei* extract during 21 days was effective in improving the oxidative state of both the brain and liver tissues of 21 months old rats. Treatment with the extract, for example, considerably increased the total antioxidant capacity of the brain tissue and diminished lipid peroxidation and the levels of reactive oxygen species in both brain and liver [4,5]. Treatment with the extract was also effective in improving the impaired energy metabolism of brain mitochondria from old rats, especially the coupled respiration driven by succinate [4]. These actions have been interpreted as resulting, partly at least, from the antioxidant activity of the *A. blazei* extract [4,5], which is quite pronounced [6]. The *A. blazei* extract contains in fact several phenolics such as gallic acid, syringic acid and pyrogallol, which have been demonstrated to possess high antioxidant activities [7]. The *A. blazei* extract is also rich in polysaccharides [8], a fact that can be significant if one remembers that fungal polysaccharides have been recently demonstrated to exert hepatoprotective actions [9,10]. Furthermore, *A. blazei* is also rich in nucleotides and nucleosides [11], as adenosine, for example, which like other activators of A_1_ purinergic receptors, confers cytoprotection in the cardiovascular and central nervous systems by activating cell surface adenosine receptors [12,13]. Activation of these receptors, in turn, is postulated to activate antioxidant enzymes via protein kinase C phosphorylation of the enzymes or of intermediates that promote activation [12].

The observation that a short treatment with relatively high doses (200 mg/kg) of the *A. blazei* extract improves the oxidative status of the brain and liver in old rats [4,5] raises the question if it is possible to avoid an unfavorable oxidative status in the old-age by administering continuously lower doses during the whole aging process. To find an answer to this question was precisely the purpose of the present work, in which rats were treated continuously with doses of 50 mg/kg of the *A. blazei* aqueous extract starting at an age of 7 months and continuing treatment until the age of 23 months. In order to obtain a more complete picture, several oxidative indicators were measured in brain and liver. The results should contribute to an understanding of the possible effects of a continuous use of *A. blazei* extracts, especially during aging.

## 2. Results and Discussion

**Figure 1:**
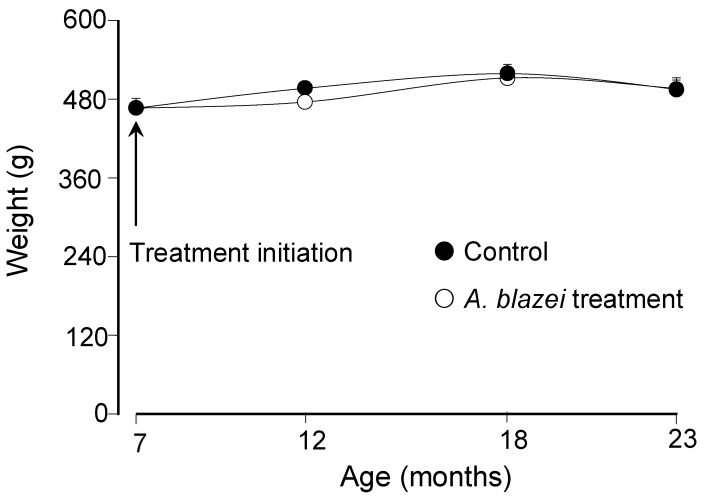
Body weight as a function of age of control and *A. blazei*-treated rats.

The body weight evolution of *A. blazei*-treated and control rats can be appreciated in Figure 1. The mean weight of the rats at the age of 7 months was 466.7 ± 14.6 gram. Figure 1 shows that the weight changed minimally with the age and also that it was not significantly affected by the *A. blazei* treatment. Since the weight varied only minimally over the whole period, the same can be said about the dosis per body weight which varied between 48.1 and 53.6 mg/kg.

**Figure 2:**
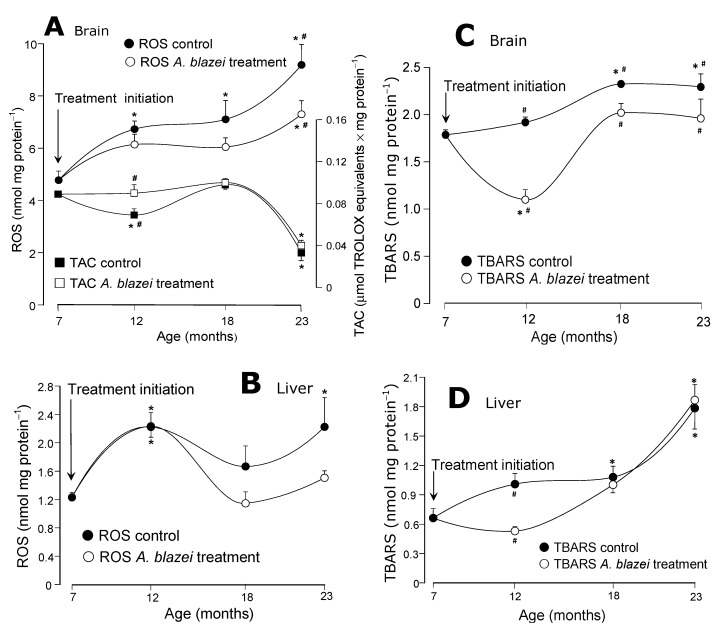
(**A**) Total antioxidant capacity (TAC) and oxygen reactive species (ROS) levels of the brain homogenate. (**B**) Total antioxidant capacity (TAC) and oxygen reactive species (ROS) levels of the liver homogenate. (**C**) Lipid peroxidation levels (TBARS) of the brain homogenate. (**D**) Lipid peroxidation levels (TBARS) of the liver homogenate. The data points represent the means ± mean standard errors of five to seven animals. The statistical analysis consisted of MANOVA followed by Duncan’s multiple range *post hoc* testing. Data points labeled with asterisks (*) are values significantly different (*p* ≤ 0.05) from those found at 7 months (treatment initiation). Statistically different pairs of values for a given age are labeled with hashes (#).

The evolution of the reactive oxygen species (ROS) contents and the total antioxidant capacity (TAC) of the brain tissue is shown in Figure 2A. In control rats the ROS content increased progressively from the age of 7 to 23 months. The increase from 7 to 23 months was actually equal to 93%. In *A. blazei*-treated rats the ROS contents also tended to increase with age, but they always remained at lower levels when compared to the controls. At the age of 23 months the significant difference amounted to 21%. The total antioxidant capacity (TAC) in the brain of control rats decreased 19% at the age of 12 months; this decrease did not occur in *A. blazei*-treated rats. After this age, however, control and *A. blazei*-treated rats behaved similarly, including the pronounced drop in TAC at the age of 23 months. The ROS contents of the hepatic tissue in control rats, as shown in Figure 2B, oscillated during aging at levels that were higher than those found at the age of 7 months. In rats treated with the *A. blazei* extract the levels tended to be lower, but statistically they cannot be distinguished from those of the controls.

The evolution of the lipid peroxidation levels (TBARS) is shown in panels C and D of Figure 2. In the brain (Figure 2C) they increased until the age of 18 months in control rats. At the age of 23 months they were 30% higher than those at 7 months. In *A. blazei*-treated rats there was a relatively pronounced drop in the TBARS levels at the age of 12 months, the difference between control and treated rats amounting to 44%. During the aging process, however, this difference diminished because the TBARS levels increased again in treated rats and at the age of 23 months the difference was of only 15%. In the liver (Figure 2D) the lipid peroxidation levels also increased with age in the control rats. In the 23-months old rats the TBARS levels were 170% higher than at the age of 7 months. In *A. blazei*-treated rats the hepatic TBARS levels at the age of 12 months were close to the levels at the 7 months age and, consequently, were significantly lower than the corresponding levels in non-treated rats. With the increase in age, however, the hepatic TBARS levels of treated rats equalled those of the non-treated rats.

**Figure 3:**
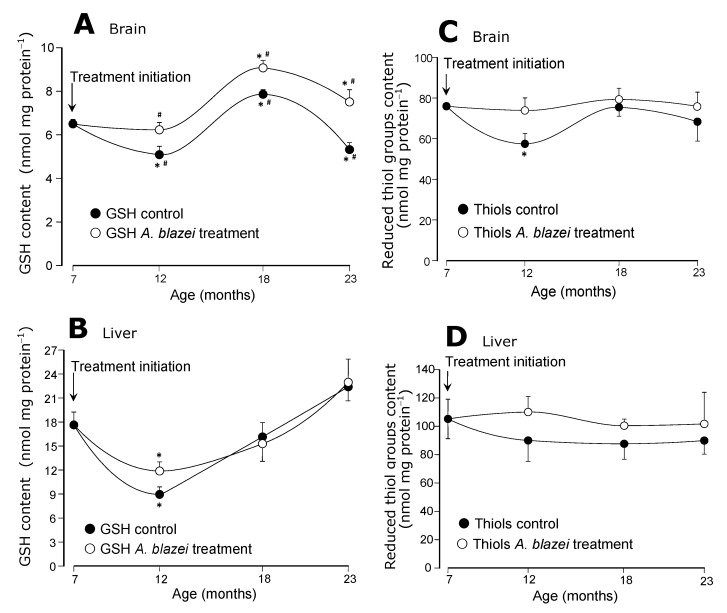
(**A**) Reduced glutathione (GSH) levels of brain homogenates. (**B**) Reduced glutathione (GSH) levels of the liver homogenates. (**C**) Protein thiol groups of the brain homogenates. (**D**) Protein thiol groups of the brain homogenates. The data points represent the means ± mean standard errors of five to seven animals. The statistical analysis consisted of MANOVA followed by Duncan’s multiple range *post hoc* testing. Data points labeled with asterisks (*) are values significantly different (*p* ≤ 0.05) from those found at 7 months (treatment initiation). Statistically different pairs of values for a given age are labeled with hashes (#).

The evolution of the GSH contents in brain and liver is shown in panels A and B of Figure 3. In the brain of control rats (Figure 3A) there was an oscillation with lower contents at the age of 12 months followed by higher contents at the age of 18 months and a final drop at the age of 23 months. The *A. blazei*-treated rats followed the same pattern, but always at a higher level. At the age of 23 months the GSH content of the brain of treated rats was 42% higher than that of non-treated rats. In the liver (Figure 3B) the GSH content did not differ in control and *A. blazei*-treated rats. The diminution that occurred at the age of 12 months when compared to the age of 7 months was similar in both groups.

The reduced thiol groups contents remained essentially the same in both treated and non-treated rats, as revealed by panels C and D of Figure 3. This is true for both brain (Figure 3C) and liver (Figure 3D) even though a drop occurred in the brain of control rats at the age of 12 months when compared to 7 months. Statistical significance relative to the contents in treated rats at the corresponding age, however, is lacking.

**Figure 4:**
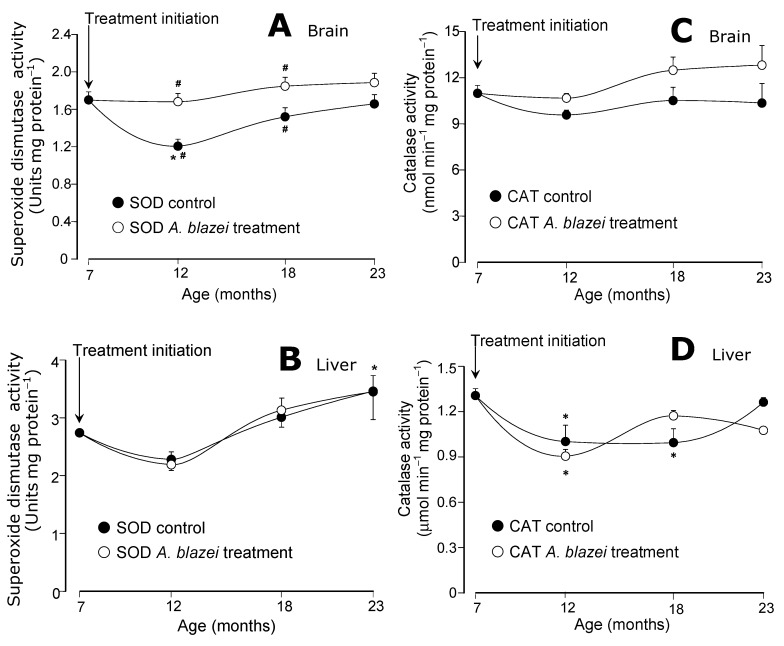
(**A**) Superoxide dismutase (SOD) activities of brain homogenates. (**B**) Superoxide dismutase (SOD) activities of liver homogenates. (**C**) Catalase (CAT) activities of brain homogenates. (**D**) Catalase (CAT) activities of liver homogenates. The data points represent the means ± mean standard errors of five to seven animals. The statistical analysis consisted of MANOVA followed by Duncan’s multiple range *post hoc* testing. Data points labeled with asterisks (*) are values significantly different (*p* ≤ 0.05) from those found at 7 months (treatment initiation). Statistically different pairs of values for a given age are labeled with hashes (#).

**Figure 5:**
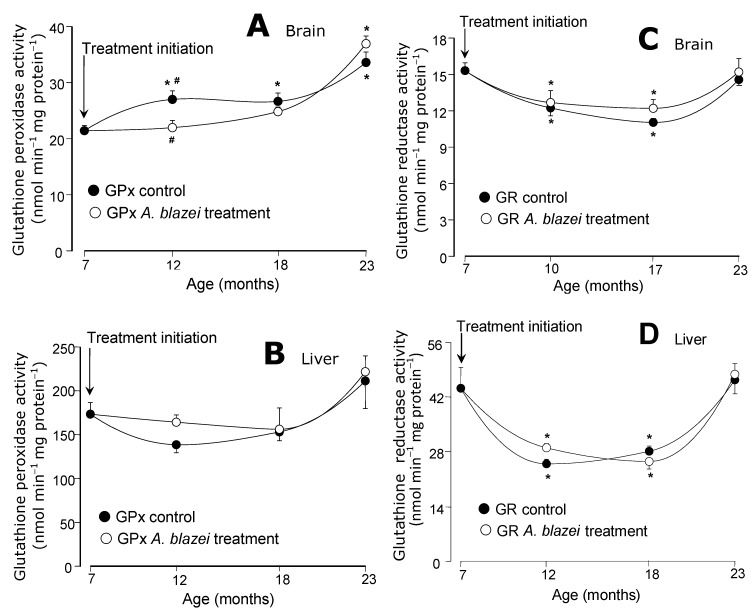
(**A**) Glutathione peroxidase (GPx) activities of brain homogenates. (**B**) Glutathione peroxidase (GPx) activities of liver homogenates. (**C**) Glutathione reductase (GR) activities of brain homogenates. (**D**) Glutathione reductase (GR) activities of liver homogenates. The data points represent the means ± mean standard errors of five to seven animals. The statistical analysis consisted of MANOVA followed by Duncan’s multiple range *post hoc* testing. Data points labeled with asterisks (*) are values significantly different (*p* ≤ 0.05) from those found at 7 months (treatment initiation). Statistically different pairs of values for a given age are labeled with hashes (#).

The evolution of the antioxidant enzymatic activities during aging is shown in Figure 4 and Figure 5. The superoxide dismutase (SOD) of the brain presented the most pronounced differences between the *A. blazei*-treated and non-treated rats. As revealed by Figure 4A the SOD activity in the brain of control rats suffered a drop at the age of 12 months when compared to 7 months. In the brain of the *A. blazei*-treated rats no such drop occurred. In consequence, at the age of 7 months, the SOD activity in the brain of *A. blazei*-treated rats was 38% higher when compared to control rats. In the non-treated rats, however, the SOD activity recovered gradually during aging. At the age of 18 months the difference amounted to only 21% and at 23 months it was no longer significant. In the liver (Figure 4B), no significant changes were detected in the SOD activity of control and *A. blazei*-treated rats. There was a tendency toward increasing activities at the age of 23 months, but the statistical significance is rather poor.

The catalase activities in the brain did not present significant changes during aging (Figure 4C) in both control and *A. blazei*-treated rats. In the liver, significant decreases were detected in both control and treated rats at the ages of 12 and 18 months, but no differences between both groups were found (Figure 4D). The glutathione peroxidase activities in the brain (Figure 5A) tended to increase with age. A significant difference between control and treated rats was found only at the age of 12 months at which non-treated rats presented a somewhat higher activity. In the liver (Figure 5B) no significant changes in the glutathione peroxidase activity were found. The glutathione reductase of both brain and liver (panels C and D in Figure 5), on the other hand, presented lower activities at the ages of 12 and 18 months, when compared to 7 months, in both control and *A. blazei*-treated rats. The decrease was more pronounced in the liver. A recovery, however, occurred in both liver and brain at the age of 23 months.

The question that was formulated in the Introduction when expliciting the purposes of the present work was whether it is possible to avoid an unfavorable oxidative status in the old-age by administering continuously lower doses of the *A. blazei* extract during the whole aging process. The question was formulated taking into account previous results of experiments in which the administration of 200 mg/kg of an *A. blazei* extract during 21 days to old rats resulted in an improved oxidative state of both brain and liver [4,5]. The idea was to replace the higher concentrations of the active principles, that were probably achieved in consequence of the administration of relatively high doses of the extract, by a more prolonged treatment with lower doses and presumably lower concentrations of the active principles provided that no cumulative concentration effects would occur. The schedule adopted in the present work, with the administration of approximately 50 mg/kg starting at the age of 7 months and continuing until the age of 23 months implied in the administration of total cumulative doses of 7.5, 16.5 and 24.0 g/kg until the ages of 12, 18 and 23 months, respectively. For comparison, the administration of 200 mg/kg during 21 days to old rats amounted to 4.2 g/kg. The total administered dose in the latter was, thus, much smaller even though it is likely that higher concentrations of the active principles were achieved because this smaller total dose was administered during a shorter period of time.

The results that were obtained are not always easy to interpret although, in principle, the question if it is possible to avoid an unfavorable oxidative status in the old-age by administering continuously lower doses during the whole aging process can be answered in a positive way. Especially in the brain the continuous treatment suceeded in maintaining lower levels of lipid peroxidation (TBARS) and higher concentrations of reduced glutathione (GSH) until the age of 23 months. The reactive oxygen species (ROS) levels in the brain increased progressively with the age but in treated rats there was a constant tendency toward lower values up to the age of 18 months, culminating with significantly lower levels at the age of 23 months. In the liver the effects were much less pronounced. Rigorously speaking only the TBARS levels were substantially decreased in the liver of treated rats and this only at the age of 12 months. It must be stressed, however, that the response of the liver to the 21-days/200 mg·kg^−1^ treatment was also much less pronounced than the response of the brain to the same treatment [4,5].

The causes for the actions of the *A. blazei* extract are probably the same as those already discussed in previous studies [4,5]: (a) the free-radical scavenging ability of several constituents of *A. blazei*, as for example the phenolics [6,7,8]; (b) the cytoprotective action of adenosine and several other purinergic agents which are also present in the *A. blazei* extracts [11] and which are believed to activate antioxidant enzymes via protein kinase C phosphorylation of the enzymes or of intermediates that promote such activation [12]; (c) the antioxidant action of the polysaccharides/β-glucans [9,10] that are present in *A. blazei* [14,15]; and (d) possibly also oligopeptides, as suggested by the antioxidant activity of an *A. blazei* oligopeptide that was recently described [16].

The observation with respect to the TBARS in the liver, *i.e.*, a maximal difference between treated and non-treated rats at the age of 12 months and smaller differences (if any) in subsequent ages, was not an isolated finding. The phenomenon of a more pronounced difference between treated and non-treated rats at the age of 12 months was also observed in the brain for the following variables: (1) the total antioxidant capacity (+24% in treated rats); (2) the lipid peroxidation levels (−42%% in treated rats); (3) the GSH content (+23% in treated rats); (4) the superoxide dismutase activity (+40% in treated rats); (5) the glutathione peroxidase activity (−19% in treated rats). All these differences either diminished or vanished (in most cases) in the subsequent ages. This seems not to be a phenomenon that can be attributed to the lower daily doses that were administered in the present study when compared to the doses administered during the short period treatment of our previous studies [4,5]. A plausible explanation, depending on experimental verification, is that during the long period of treatment the rats may have developed many adaptations or resistance to the new situation of a constant and long presence of the substances contained in the extract. Such a behavior, which can be classified as a drug-resistant state, has been often described in the literature [17,18]. The phenomenon can be related to specific responses in terms of gene expressions for factors involved in the aging process per se as well as responses related to the transformation and handling of the various active components of the *A. blazei* extract.

## 3. Experimental Section

### 3.1. Preparation of the Agaricus blazei Extract

The previously grounded dehydrated basidioma of *Agaricus blazei* were submitted to an aqueous extraction as described previously with minor modifications [6]. Fruiting bodies (basidiocarps) of *A. blazei* were obtained from a local producer in Maringá, PR, Brazil, in Spring 2009. The dried basidiocarps were milled until obtaining a fine powder. The samples (10 g) were extracted by stirring with water (100 mL, 28 °C) at 130 rpm for 3 h and filtered through Whatman filter paper 1. The extraction was repeated three times. The filtrates (yield 50%) were lyophilized and stored in freezer until use.

### 3.2. Animals and Treatment

Male Wistar rats were kept in polypropylene cages (four animals per cage), with light and dark cycles of 12 h and at a temperature of 22 ± 2 °C. All rats had free access to water and were fed *ad libitum* with a standard laboratory diet (Nuvilab-Nuvital^®^, Colombo, Paraná, Brazil). Treatment with the *A. blazei* extract was initiated when the rats attained the age of seven months. The treatment consisted in the daily intragastric administration of 25 mg (50 mg/kg) of the freeze-dried extract suspended in water. Water was administered to control rats. Analyses of control and *A. blazei*-treated rats were carried out at the following ages: (1) 7 months (zero time of the *A. blazei* treatment); (2) 12 months; (3) 18 months; (4) 23 months. All experiments were done in accordance with the internationally accepted recommendations in the care and use of animals and the protocol was approved by the Ethics Committee for Animal Experimentation of the University of Maringá (protocol 033/2010).

### 3.3. Preparation of the Brain and Liver Homogenates

Rats were starved for 18 h and then anesthetized by intraperitoneal injection of thiopental (50 mg/kg). The criterion of anesthesia was the lack of body or limb movement in response to a standardized tail clamping stimulus. The brain and liver of each rat were surgically removed with scissors, clamped with liquid nitrogen and stored at temperatures under −150 °C. The tissue suspensions (10% w/v in 0.1 M phosphate buffer, pH 7.4) were homogenized by means of a van Potter-Elvejhem homogenizer. Protein contents were determined with the Folin phenol reagent [19] using bovine-serum albumin as standard.

### 3.4. Determination of the Total Antioxidant Capacity (TAC)

The total antioxidant capacity of the brain was determined colorimetrically with 2,2′-azino-bis(3-ethylbenzo-thiazoline-6-sulphonic acid (ABTS; [20]). Aliquots (50 µL) from the supernatant of a 10,000*× g* centrifugation of the brain homogenate were added to 1.8 mL of 0.4 M acetate buffer (pH 5.8) plus 150 µL of a cationic ABTS solution (30 mM acetate buffer, pH 3.6, containing 10 mM ABTS and 4 mM H_2_O_2_). After 5 min of incubation in the dark, the absorbance at 734 nm was read against water. The compound 6-hydroxy-2,5,7,8-tetramethylchroman-2-carboxylic acid (Trolox) was used as a standard and the results were expressed as μmol Trolox equivalents per mg protein.

### 3.5. Determination of Lipid Peroxidation, Reduced Glutathione and Protein Reduced Thiol Contents

The levels of lipid peroxidation were measured in the brain and liver homogenates by means of the TBARS method (thiobarbituric reactive substances). The concentration of lipoperoxides was determined spectrophotometrically at 532 nm using an extinction coefficient (ε_532nm_) of 1.56 × 10^5^ M^−1^·cm^−1^. The results were expressed as nmol malondialdehyde (MD) per mg protein [21].

The reduced glutathione (GSH) levels of liver and brain homogenates were determined spectrofluorimetrically [22]. Aliquots (60 µL) of the brain or liver homogenates were added to the reaction medium (1.0 mL) containing 125 mM sucrose, 65 KCl and 10 mM HEPES (pH 7.4). After protein precipitation with 13% trichloroacetic acid, 100 µL of the supernatant were added to 2.0 mL of a medium containing 0.1 M NaH_2_PO_4_ and 5 mM EDTA, pH 8.0. One hundred mL of 1 mg/mL *o*-phthalaldehyde was added and, after 15 min at room temperature, the fluorescence intensity was determined (excitation: 350 nm; emission: 420 nm). Standards were run in parallel and the glutathione concentration was expressed as µmol per mg protein.

The reduced protein thiol groups in the brain and liver homogenates were determined using the compound 5,5′-dithiobis 2-nitrobenzoic acid (DTNB) [23]. Proteins in 100 µL of the homogenate were precipitated with 1.0 mL 5% trichloroacetic acid + 5 mM EDTA. After centrifugation at 2000 × *g* for 3 min, the precipitate was homogenized with a pellet homogenizer. The process of precipitation/homogenization was repeated twice and the final precipitate was suspended in 3.0 mL of 0.1 M TRIS buffer (pH 7.4) containing 5 mM EDTA and 0.5% sodium dodecyl sulfate. An aliquot of 400 µL of this solution was transferred to 1.6 mL of 0.1 M TRIS buffer (pH = 8.6) containing 5 mM EDTA with a further addition of 20 µL of 10 mM DTNB. After 10 min in the dark, the absorbance against blank was determined at 412 nm. The blank consisted in 2.0 mL 0.1 M TRIS buffer (pH = 8.6) plus 20 µL of 10 mM DTNB. The concentration of reduced thiols was calculated using a molar extinction coefficient of 1.36 × 10^4^ M^−1^·cm^−1^ and expressed as nmol per mg protein.

### 3.6. Reactive Oxygen Species (ROS) Determination

The levels of reactive oxygen species were estimated in aliquots from the supenatants of the 10,000 × *g* centrifugation of the brain and liver homogenates using the reaction with 2′,7′-dichlorofluorescein diacetate [24]. The samples were incubated with 100 mM 2′,7′-dichlorofluorescein diacetate (DCFH-DA) during 30 min at 37 °C. The reaction was stopped in bath ice and the formation of oxidized 2′,7′-dichlorofluorescein (DCF) was measured fluorimetrically with excitation at 504 nm and emission at 529 nm. The ROS content was calculated using a standard curve with H_2_O_2_ and the results were expressed as nmol mg protein^−1^. All steps were processed in the dark and a blank containing DCFH-DA was used to exclude autofluorescence.

### 3.7. Determination of Antioxidant Enzymes

The activity of catalase (CAT) was evaluated by measuring spectrophotometrically the decomposition of H_2_O_2_ at 240 nm [25]. Aliquots from the supernatants of the 10,000*× g* centrifugation of either liver or brain homogenate (≈0.15 mg protein/mL) were added to a solution containing 50 mM TRIS (pH 8.0), 0.25 mM EDTA and 30 mM H_2_O_2_. The drop in absorbance during the first minute of incubation was measured at 25 °C. A standard H_2_O_2_ curve was used to calculate the enzyme activity, which was expressed as μmol min^−1^ mg protein^−1^.

The activity of the superoxide dismutase (SOD) was assayed by its capacity to inhibit the auto-oxidation of pyrogallol in alkaline medium which was monitored spectrophotometrically at 420 nm [26]. One unit of SOD is defined as the amount of enzyme promoting 50% inhibition of pyrogallol auto-oxidation. Aliquots from the supernatants of the 10,000 × *g* centrifugation of either brain homogenate or liver homogenate (≈0.35 mg protein/mL) were added to solutions containing 0.2 M TRIS (pH 8.2) and 2 mM EDTA. The reaction was started by adding 0.1 mM pyrogallol. The change in absorbance was monitored, the initial rate computed and the activity expressed as SOD units per mg protein.

The activity of glutathione peroxidase was determined as the decrease in absorbance at 340 nm due to NADPH oxidation dependent on H_2_O_2_ at 25 °C [27]. Aliquots from the supernatants of the 10,000*× g* centrifugation of liver or brain homogenate (≈0.4 mg protein/mL) were added to a solution containing 40 mM phosphate buffer (pH 7.0), 0.5 mM EDTA, 1.0 mM sodium azide, 1.0 mM reduced glutathione, 1.5 mM NADPH and 2 units of glutathione reductase. The reaction was initiated by the addition of H_2_O_2_ (0.2 mM) and monitored during 90 s. The initial rates were obtained by extrapolation to zero time and the activity computed as nmol min^−1^ mg protein^−1^ using the molar extinction coefficient of NADPH (6.22 × 10^3^ M^−1^·cm^−1^).

The activity of glutathione reductase (GR) was determined as the decrease in absorbance at 340 due to the NADPH oxidation [28]. Aliquots from the supernatants of the 10,000 × *g* centrifugation of brain homogenate or liver homogenate (≈0.6 mg protein/mL) were added to 1 mL of a solution containing 50 mM phosphate buffer (pH 8.0), 2 mM EDTA, 0.15 mM NADPH and 0.5 mM oxidized glutathione (GSSG) at 25 °C. The initial rates were obtained by extrapolation to zero time and the activity computed as nmol min^−1^ mg protein^−1^ using the molar extinction coefficient of NADPH (6.22 × 10^3^ M^−1^·cm^−1^).

### 3.8. Statistics

All results are presented as means ± mean standard errors. Evaluation of the statistical significance was done by means of multivariate variance analysis (MANOVA) followed by *post hoc* Duncan’s multiple range testing. The 5% level (*p* < 0.05) was adopted as the significance criterion.

## 4. Conclusions

It can be concluded that aqueous extracts of *A. blazei* are able to stimulate the body defenses against oxidative stress during aging. However, if one compares the effects of short period treatments [4,5] with the results obtained in the present study, it can also be suggested that the best therapeutic schedule would not be a continuous intake of an *A. blazei* preparation but rather an intermittent treatment with ten or twenty day pulses. Pulsed intermittent therapy is a widespread procedure for preventing a drug resistance state [17,18]. If this really applies to the *A. blazei* treatment employed in the present study, however, still depends on further and certainly very intensive research. In particular, clinical studies, as those already done for the immunomodulatory properties and others [3], are highly desirable.
